# Gankyrin Drives Malignant Transformation of Gastric Cancer and Alleviates Oxidative Stress via mTORC1 Activation

**DOI:** 10.1155/2018/9480316

**Published:** 2018-10-21

**Authors:** Bo Huang, Weiyang Cai, Qian Wang, Feng Liu, Ming Xu, Yanjie Zhang

**Affiliations:** Department of Oncology, Shanghai 9th People's Hospital, Shanghai Jiao Tong University School of Medicine, 280 Mohe Road, Shanghai 201999, China

## Abstract

Gastric cancer, as a malignant epithelial tumor, is a major health threat leading to poor overall survival and death. It is usually diagnosed at an advanced stage due to asymptomatic or only nonspecific early symptoms. The present study demonstrated that gankyrin contributes to the early malignant transformation of gastric cancer and can be selected to predict the risk of gastric cancer in those patients harboring the precancerous lesions (dysplasia and intestinal metaplasia). In addition, a new insight into gastric cancer was provided, which stated that gankyrin alleviates oxidative stress via mTORC1 pathway activation. It can potentiate the mTORC1 by PGK1-AKT signaling that promotes the tumor process, and this phenomenon is not completely consistent with the previous report describing colorectal cancer.

## 1. Introduction

Gankyrin (also named p28, p28GANK, or PSMD10) acts as a molecular chaperone during the assembly of the 26S proteasome, specifically the 19S regulatory complex [1–3], and is commonly overexpressed in human malignancies [4–9]. It is involved in the negative regulation of pRB1 and p53/TP53 to execute the oncoprotein function [3, 10]. The competitive binding with CDKN2A triggers gankyrin to regulate the CDK4-mediated phosphorylation and further proteasomal degradation of RB1 [11, 12]. Similarly, gankyrin binds to MDM2, a major E3 ubiquitin ligase for p53, and increases the MDM2-mediated mono- or polyubiquitination of p53 in order to exhibit the antiapoptotic activity in the cells that were injured by DNA-damaging agents [4, 10, 12, 13]. Moreover, oxidative stress disorder is also speculated to be involved in the development of various human diseases including cancer [14, 15]. In hepatocellular carcinoma, overexpressed gankyrin inhibits the proteasomal degradation of Nrf2 by blocking the interaction between Nrf2 and Keap1 and plays an antioxidative role via the feedback regulation of Nrf2 [15]. Beyond the function of mediating protein degradation, gankyrin can directly bind to the NF-*κ*B component RelA and accelerate its chromosomal region maintenance-1- (CRM-1-) mediated nuclear export in hepatocellular carcinoma [16]. This phenomenon might be attributed to the gankyrin-mediated attenuation of the acetylation of RelA and its retention in the cytoplasm to suppress NF-*κ*B transactivation [17]. This evidence reveals that gankyrin is involved in the multiple biological and physiological processes in cells and contributes to the development of cancer. The most recent data from our group showed that gankyrin mediates TSC2 for degradation and regulates mTOR signaling via a p53-independent pathway in colorectal cancer [18]. However, whether gankyrin plays an analogical function or regulatory role in the cell signaling pathway in gastric cancer is yet an enigma.

Gastric (stomach) cancer is the third leading cause of deaths from cancer accounting for 7% of the cases and 9% deaths after lung and liver cancer [19]. It is primarily caused by *Helicobacter pylori* infection, which accounts for >60% of the cases [20, 21], smoking [21], diet [22], and genetics [23, 24]. Due to the absence of early typical clinical signs, gastric cancer has often been diagnosed at an advanced stage and may have occurred as distant metastasis before the symptoms onset. These manifestations might be the primary cause of the relatively poor prognosis of the disease [25]. However, increasing evidence has revealed that gankyrin is a promising target for the diagnosis and treatment of several cancers [8, 26–29]. Nevertheless, compelling proofs to describe and illustrate the functional role or clinical significance of gankyrin in the development of gastric cancer are yet lacking.

Gastric intestinal metaplasia (GIM) and dysplasia, precursor lesions to and gastric cancer, are usually observed in the milieu of long-standing nonatrophic gastritis (NAG) and chronic atrophic gastritis (CAG) [30, 31]. Herein, we demonstrated that gankyrin is transcriptionally activated in tissue cells since patients harbored chronic atrophic gastritis, precancerous lesion (GIM and dysplasia), or gastric cancer and, thus, might be a preeminent candidate target for the early diagnosis of gastric cancer. In addition, we also found that gankyrin restricted the oxidative stress by stimulating the mTORC1 signaling in gastric cancer.

## 2. Materials and Methods

### 2.1. Tissues and Immunohistochemistry

Deidentified tissues from 262 patients were included. 77 malignant infiltrating gastric cancer tissues and paired noncancerous tissues were collected from the hospital and developed into tissue array by OUTDO Biotech (Shanghai, China). 120 noncancerous tissues, including nonatrophic gastritis (NAG), chronic atrophic gastritis (CAG), CAG with intestinal metaplasia (IM), and CAG with dysplasia (dys) gastric tissue, were acquired by endoscopy, 30 cases for each group. 65 gastric cancer samples with complete follow-up data were collected for survival analysis. Follow-up time and survival time were calculated from the day of the operation to the end of the follow-up or the date of death due to recurrence and metastasis. The study protocols were approved by the SJTUSM (Shanghai Jiao Tong University School of Medicine) Ethics Committee. All procedures adhere to the BRISQ Guidelines reporting research on human biospecimens. Immunohistochemical detection of gankyrin was performed using a streptavidin-biotin complex method as described previously [18]. For quantitative analysis, a histoscore (H-score) was calculated using Aperio Scan Scope systems (Vista, CA, USA) as previously described, by multiplying the intensity score and the fraction score, producing a total range of 0–300 [18]. Tissue sections were examined and scored separately by two independent investigators blinded to the clinicopathologic data.

### 2.2. Cell Culture and Reagents

The gastric cancer cell lines, MKN45 and MKN74, were purchased from the Shanghai Institute for Biological Sciences (SIBS, Shanghai, China) and cultured in RPMI 1640 medium (HyClone, Los Angeles, CA, USA) containing 10% fetal bovine serum (FBS; HyClone) and 100 U/mL penicillin/streptomycin under conditions of 5% CO_2_ and humidified air at 37°C. The lentiviral pCDH-EF1-MCS-T2A-copGFP gankyrin plasmid was constructed, packed, and purified by Sunbio (Shanghai, China).

### 2.3. MTT Assay

The activity of MKN45 and MKN74 cells overexpressing gankyrin or vector control (NC) was determined by MTT assay. Briefly, the cells were seeded in quintuplicate in 96-well culture plates and cultured for up to 96 h, followed by an addition of 20 *μ*L of 5 mg/mL MTT (3-(4,5-dimethyl-2-thiazolyl)-2,5-diphenyl-2-H-tetrazolium bromide; Sigma-Aldrich, St. Louis, MO, USA) solution per well. After incubation for 4 h at 37°C, the supernatant was replaced with 100 *μ*L DMSO. The absorbance per well was measured by a Microculture Plate Reader at 570 nm and 630 nm after 20 min agitation at room temperature. The data represent the means ± standard deviation (SD) from three independent triplicate experiments.

### 2.4. Colony Forming Assay

Cells were seeded in triplicate in 12-well plates to form colonies for up to 7–10 days. The medium containing selective antibiotics was replaced every 3–5 days. The colonies were stained with methylene blue and counted. Data represent the means ± SD from three independent experiments performed in triplicate.

### 2.5. Soft Agar Assay

The anchorage-independent growth of MKN45 and MKN74 cells overexpressing gankyrin or NC was determined by soft agar assays. Briefly, 0.7% basal-layer agar was prepared with 1.4% low-melting-point agarose and 2 × cell medium (1 : 1, *v* : *v*). 1 mL of basal layer agar was added to each well of a 6-well plate. The exponentially growing cells were harvested by trypsinization to 5000 cells/mL single-cell suspension. 0.35% top-layer agar was prepared with 0.7% low-melting point agarose and 2 × cell medium (1 : 1, *v* : *v*). Subsequently, 1 mL top-layer agar was blended with 100 *μ*L single-cell suspension/well (500 cells/well). The cells were incubated for up to 1–2 weeks at 37°C after solidification at room temperature. Cultures were stained with p-iodonitrotetetrazolium violet (Sigma-Aldrich) for 2 h and then inspected and photographed using a MiniCount Colony Counter. The colonies containing more than 50 cells were counted and imaged. The data represent the means ± SD from three independent experiments in triplicate.

### 2.6. Transwell Invasion Assay

The cell suspension was prepared in a blank culture medium containing 5 × 10^5^ cells/mL for 24-well invasion chambers. The upper surface of the membrane was scrubbed carefully with a cotton swab to remove the remaining cells, and Matrigel matrix after chambers was incubated for up to 16–18 h at 37°C. The cells on the lower surface of the membranes were fixed with 100% methanol and stained with 0.5% crystal violet. The invaded cells were imaged and counted in several fields under the microscope at approximately 40x–100x magnification. Data represent the means ± SD from three independent triplicate experiments.

### 2.7. Immunoblotting

Total protein was extracted by RIPA lysis Buffer (Sigma-Aldrich) and subjected to immunoblotting as described previously [18]. Reagents were obtained from the following sources: antibodies for gankyrin (Santa Cruz, Dallas, Texas, USA); antibodies for PGK1 (Abcam, Cambridge, MA, USA); antibodies for p-S6K (T398), S6K1, S6, p-S6 (S235/236), mTOR, p-AKT (S473), AKT, p-4E-BP1 (T37/46), and 4E-BP1; and HRP-conjugated secondary antibody (Cell Signaling Technology; Danvers, MA, USA).

### 2.8. Reactive Oxygen Species (ROS) Fluorescent Probe

MKN45 cells overexpressing gankyrin or NC were treated with DMSO or 10 nM rapamycin for 1 h. Oxidative stress of treated cells was determined by 2′,7′-dichlorodihydrofluorescein diacetate (H2DCF-DA) and dihydroethidium (DHE) that indicate the level of ROS. DHE or H2DCF-DA probe solution was diluted to an appropriate concentration by culture medium, and the cell culture medium was replaced by the diluted probe solution. After incubation at room temperature for 10–90 min in the dark light, the cells were washed with fresh solution and imaged by green or blue filters using a fluorescence microscope.

### 2.9. Statistical Analysis

Statistical analysis was performed with the SAS for Windows and GraphPad Prism V6 (GraphPad Prism Inc., USA); *P* < 0.05 was considered to be statistically significant. The results were expressed as the mean ± SD. The correlation between gankyrin expression and clinicopathological parameters was analyzed by Fisher's exact test. The comparisons were analyzed using Student's *t*-test. The correlation between gankyrin and PGK1 expressions was tested by Pearson's correlation analysis. The cancer-specific survival curves were estimated by Kaplan-Meier plots and log-rank test.

## 3. Results

### 3.1. Gankyrin Contributes to the Early Malignant Behavior of Gastric Cancer

**Figure 1:**
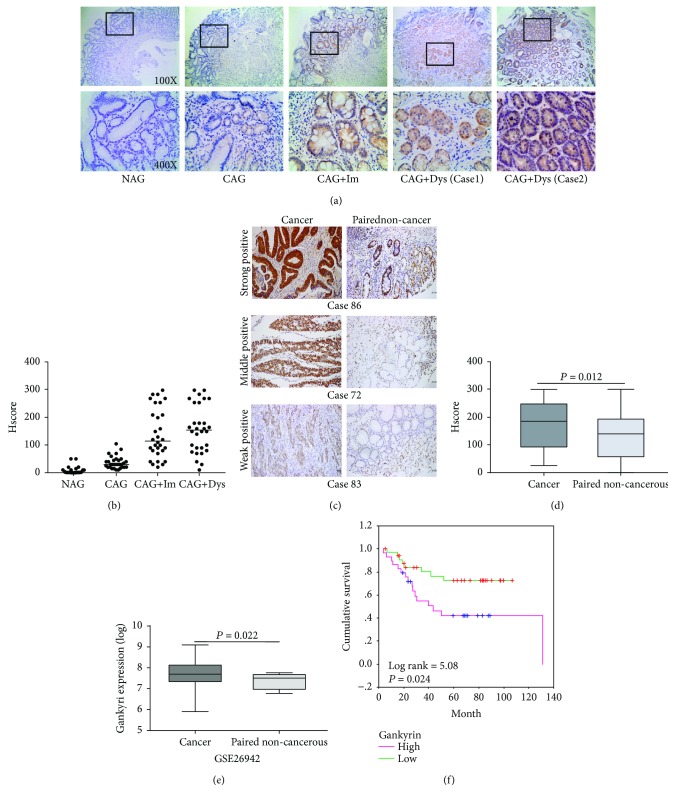
Gankyrin contributes to the early malignant transformation of gastric cancer. (a) Immunohistochemistry (IHC) staining of gankyrin in noncancerous and precancerous gastric tissue sections. Representative images of stained nonatrophic gastritis (NAG), chronic atrophic gastritis (CAG), CAG with intestinal metaplasia (IM), and CAG with dysplasia (dys) gastric tissue sections (original magnification, 100x or 400x) are shown. (b) Scatter plot showing gankyrin staining level in individual noncancerous and precancerous gastric tissue sections, 30 cases for each group. (c) IHC staining of human gastric cancer tissue and paired noncancerous tissues. Representative images of the stained tumor and paired noncancerous tissue are shown with strong, moderate, and weak positivity for gankyrin expression. (d) Box plot graph showing the statistical analysis of gankyrin expression in 77 gastric cancers and paired noncancerous tissues. (e) Gankyrin mRNA expression was significantly upregulated in gastric cancer tissues as compared to normal gastric tissues based on microarray data of GSE26942 (*n* = 217). (f) Kaplan-Meier survival analysis of gastric cancer cases divided into two groups by the median value (H-score = 145) for gankyrin staining. The *P* value was calculated by the log-rank test.

To explore the relationship between gankyrin and the risk of harboring gastric cancer, we investigated the expression of gankyrin in a large panel of gastric precancerous and cancerous clinical samples. Compared to nonatrophic gastritis (NAG) tissue, the expression of gankyrin was elevated in chronic atrophic gastritis (CAG) and significantly higher in CAG with intestinal metaplasia (CAG + Im) or dysplasia (CAG + dys) (Figures 1(a) and 1(b) and Table 1). The gankyrin staining-positive rate and median H-score in dysplasia (positive rate = 90.00%, median H-score = 150) were similar to those in gastric cancer tissues (positive rate = 92.78%, median H-score = 145). Since the CAG-metaplasia/dysplasia-cancer sequence represents the process by which most gastric cancers arise, the data indicated that gankyrin overexpression is involved in the very early stage of gastric carcinogenesis; this finding was consistent with that in human colorectal precancerous and cancerous lesions [18]. In tissue array analysis, the overall gankyrin staining was stronger in tumors (median H-score = 185) as compared to the paired noncancerous tissues (median H-score = 140) (Figures 1(c) and 1(d)). This phenomenon was further validated in the published dataset (GEO access number: GSE26942), wherein gastric cancer tissues showed significantly higher gankyrin mRNA level as compared to the noncancerous tissues (*P* = 0.022) (Figure 1(e)). In the tissue array comprising 77 cases, gankyrin overexpression was associated with lymph node metastasis (*P* = 0.019), distant metastasis (*P* = 0.013), and vascular invasion (*P* = 0.037) (Table 2). The log-rank test revealed that high gankyrin expression was significantly correlated with poor survival (*n* = 65, *P* = 0.024) (Figure 1(f)). Taken together, gankyrin contributes to the early malignant behavior of gastric cancer.

### 3.2. Gankyrin Promotes the Oncogenic Properties of Gastric Cancer Cell

**Figure 2:**
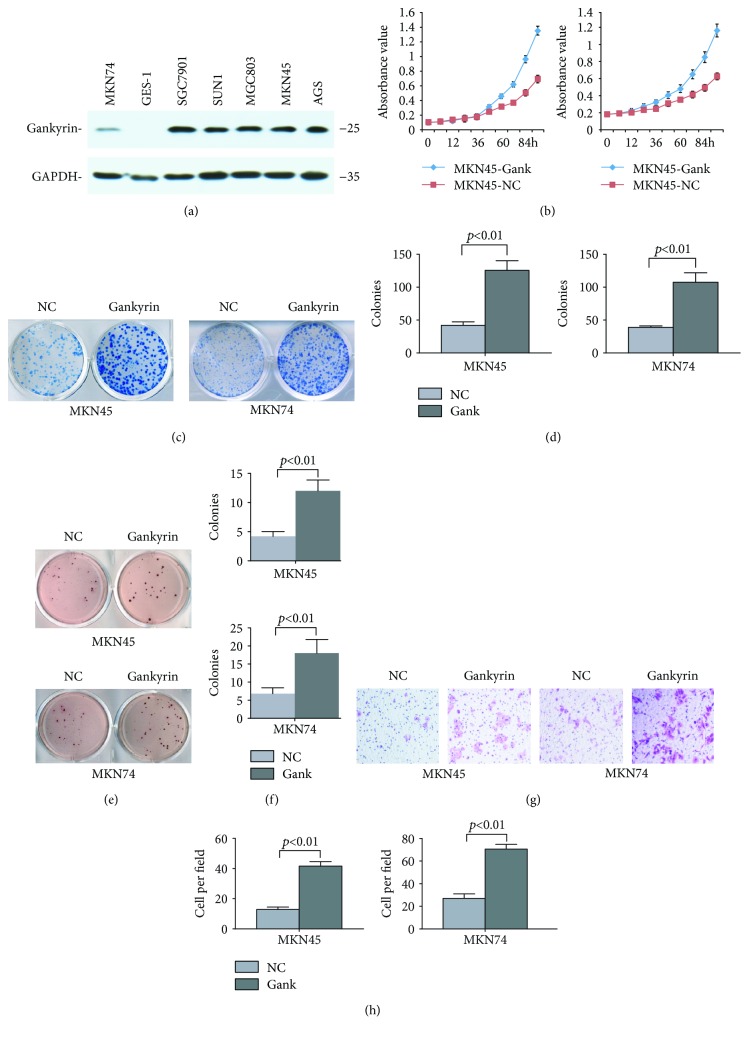
Gankyrin promotes the oncogenic properties of gastric cancer cell. (a) The basal level of gankyrin in different cell lines was detected by Western blot analysis. (b) The viability of MKN45 and MKN74 cells stably expressing gankyrin or vector control (NC) was analyzed by MTT assay. Absorbance was measured using a microculture plate reader at 490 nm. (c) MKN45 (upper panel) and MKN74 (lower panel) cells overexpressing gankyrin (-Gank) or carrying a control vector (-NC) were assessed for focus formation. The colonies were stained with methylene blue and enumerated by Image-Pro Plus (Media Cybernetics). Representative images are shown. (d) Quantification of focus formation in the experiment in (c). Data represent the means ± SD from three independent experiments in triplicate. (e) MKN45 (upper panel) and MKN74 (lower panel) cells overexpressing gankyrin (-Gank) or carrying a control vector (-NC) were plated in soft agar to determine anchorage-independent growth. The colonies were stained with p-iodonitrotetrazolium violet and enumerated by Image-Pro Plus (Media Cybernetics). Representative images are shown. (f) Quantification of soft agar colony formation in (e). Data represent the means ± SD from three independent experiments in triplicate. (g) Transwell assay was used to measure the cell invasion of MKN45 (upper panel) and MKN74 (lower panel) cells overexpressing gankyrin (-Gank) or a control vector (-NC). (h) Quantification of cell migration in (g). Data represent the means ± SD from three independent experiments in triplicate.

To inspect the phenotypes induced by gankyrin in gastric cancer cells, we firstly identified the basal levels of human normal gastric epithelial cell (GSE-1) and several gastric cancer cell lines using Western blot analysis and the results revealed that high gankyrin expression was observed in all cancer cell lines, but it was undetectable in GES-1 (Figure 2(a)). To explore the function of gankyrin in gastric cancer development, MKN74 (low-gank expression) and MKN45 (high-gank expression) cells were chosen to establish the gankyrin overexpression cell lines. The MTT assays showed that overexpressed gankyrin significantly accelerated the cell growth as compared to that by background expression (Figure 2(b)). Both foci formation assay (Figures 2(c) and 2(d)) and soft agar assay (Figures 2(e) and 2(f)) measured the anchorage-dependent or anchorage-independent cell growth and revealed that gankyrin promoted the characteristics of transformed cells with marked differences in either MKN74 or MKN45 cells. To assess the gankyrin-mediated invasion ability of gastric cancer cell, the Transwell trails showed that gankyrin significantly facilitated the motility and invasiveness of cancer cells as compared to the negative controls (Figures 2(g) and 2(h)). Taken together, gankyrin possesses the oncogenic properties to promote the malignant behavior of gastric cancer cells.

### 3.3. Gankyrin Potentiates mTORC1 Signaling via PGK1/AKT

**Figure 3:**
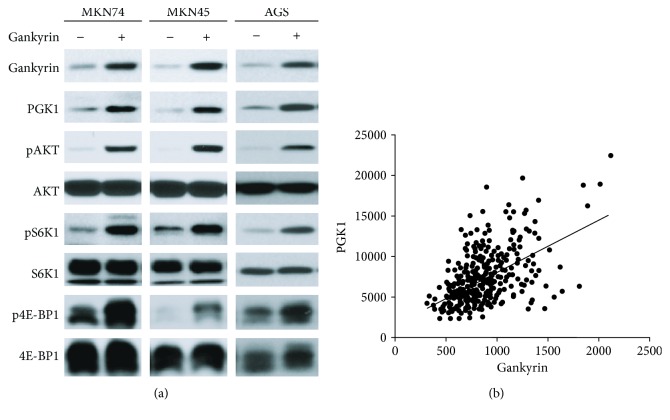
Gankyrin potentiates mTORC1 signaling via PGK1/AKT. (a) Overexpressed gankyrin activates AKT/mTORC1 signaling in gastric cancer cell lines, MKN45, MKN74, and AGS. The protein levels of PGK1, pAKT, AKT, pS6 K1, S6 K1, p4E-BP1, and 4E-BP1 were analyzed by immunoblotting. (b) The correlation plot of gankyrin and PGK1 mRNA level in 414 gastric cancer samples was presented by analyzing the TCGA cancer genome database.

In a previous report, we showed that gankyrin significantly enhanced the mTOR activity in colorectal cancer (CRC) through targeting TSC2 for degradation, independent of AKT signaling [18]. Intriguingly, gankyrin can also activate the mTORC1 signaling pathway with enhanced the levels of phosphorylated S6K1 and 4E-BP1 in gastric cancer cells (Figure 3(a)). Unlike that in CRC, gankyrin activated the AKT signaling and its upstream regulator PGK1 in gastric cancer as assessed by Western blot analysis (Figure 3(a)); this phenomenon was in agreement with the studies, wherein PGK1 activates AKT/mTOR in lung cancer [32] and regulates autophagy to promote tumorigenesis via the mTOR pathway [33]. Moreover, the mRNA level of gankyrin and PGK1 was found to be correlated in 414 gastric cancer samples according to the TCGA cancer genome database by Pearson's correlation analysis (Figure 3(b)). Taken together, gankyrin can potentiate the mTORC1 signaling via a PGK1-AKT pathway in gastric cancer.

### 3.4. Gankyrin Alleviates Oxidative Stress in Gastric Cancer Cell by Activating mTORC1

**Figure 4:**
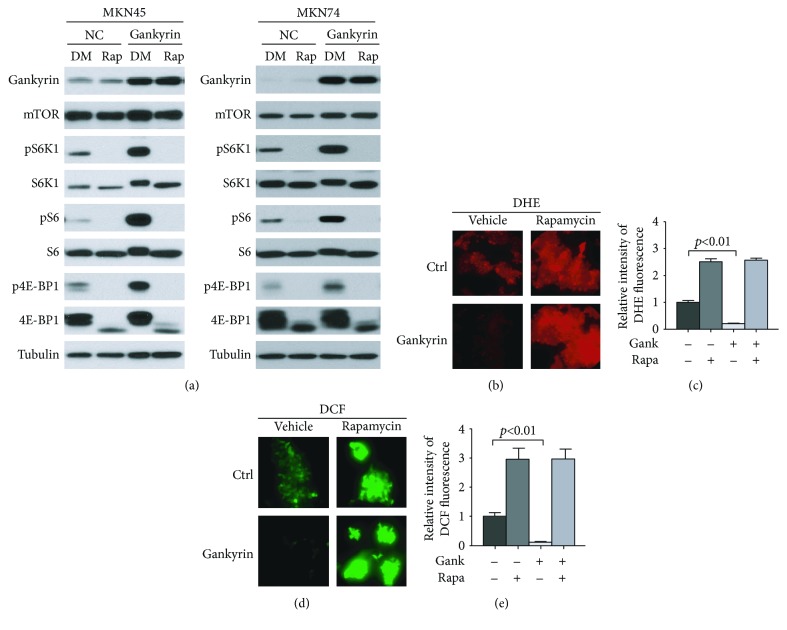
Gankyrin alleviates oxidative stress in gastric cancer cell by activating mTORC1. (a) Both MKN45 and MKN74 cells with or without overexpressed gankyrin were treated with DMSO or 10 nM rapamycin for 1 h. The protein levels of pS6 K1, S6 K1, pS6, S6, p4E-BP1, and 4E-BP1 were analyzed by immunoblotting. (b) Oxidative stress was determined by measuring reactive oxygen species (ROS) levels using dihydroethidium (DHE) staining (red) in living cells. Representative images are shown (cell number > 100). Scale bars = 10 *μ*m. (c) Quantification of fluorescence intensity in the experiment in (b). Data represent the means ± SD from three independent experiments in triplicate. *P* < 0.01 represents a significant difference. (d) Oxidative stress was determined by measuring the levels of ROS using 2′,7′-dichlorodihydrofluorescein diacetate (H2DCF-DA) staining (green) in living cells. Representative images are shown (cell number > 100). Scale bars = 10 *μ*m. (e) Quantification of fluorescence intensity in the experiment in (d). Data represent the means ± SD from three independent experiments in triplicate. *P* < 0.01 represents a significant difference.

Oxidative stress may be the cause of direct damage to DNA and, therefore, mutagenic [34]. It may also suppress apoptosis and promote cancer cell proliferation, invasiveness, and metastasis [34]. The production of reactive oxygen and nitrogen species increased by *Helicobacter pylori* infection in the stomach is also crucial for the development of gastric cancer [25, 35]. To investigate whether gankyrin affected the process of oxidative stress, the mTORC1 signaling was successfully suppressed using rapamycin in both MKN45 and MKN74 cells with or without gankyrin overexpression (Figure 4(a)). Representative DHE staining and quantification data showed that overexpressed gankyrin significantly inhibited the reactive oxidative species (ROS) as compared to the negative control (Figures 4(b), 1^st^ and 3^rd^ panels, and 4(c)). However, ROS was sustained by rapamycin treatments either with or without gankyrin overexpression (Figures 4(b), 2^nd^ and 4^th^ panels, and 4(c)), which was in agreement with DCF staining and quantification data (Figures 4(a) and 4(e)). Altogether, gankyrin can alleviate the oxidative stress by activating mTORC1 in the gastric cancer cell.

## 4. Discussion

Gastric cancer, as a malignant epithelial tumor, continues to be a major health threat related to death and poor overall survival > 5 years in both sexes worldwide [25]. In the early stages, gastric cancer either is often asymptomatic or causes only nonspecific symptoms, which might be associated with indigestion, abdominal discomfort, anorexia, or a burning sensation. Without the endoscope detection, the occurrence of gastric cancer at an early stage cannot be diagnosed easily for a timely treatment [25]. Gastric carcinogenesis is a multistep process that develops from chronic gastritis, atrophy, gastric intestinal metaplasia (GIM), and dysplasia and finally leads to gastric cancer [36]. GIM and dysplasia are speculated as the premalignant stage of gastric cancer in a population of patients [37, 38], guiding the appropriate clinical recommendations for reducing the risk of gastric cancer [39].

Herein, we revealed that Gankyrin, an oncoprotein and a potential therapeutic target in multiple cancer diseases [7, 8, 18, 24, 26, 27], was a great biomarker for the early diagnosis of gastric cancer according to the detection and analysis of its expression in a large cohort of gastric precancerous and cancerous clinical samples (Figure 1). The significantly enhanced expression of gankyrin in CAG with intestinal metaplasia or dysplasia indicated an increased risk of gastric carcinoma. Moreover, high level of gankyrin expression in gastric tumor samples or cell lines demonstrated its role in promoting the process of gastric cancer, including tumor growth, metastasis, or vascular invasion *in vivo* (Figure 1) and *in vitro* (Figure 2); also, it was significantly correlated with poor survival in clinics (Figure 1(f)). In addition to environmental risk factors (infection, smoking, or diets), a number of molecular and genetic aberrations also contribute to gastric carcinogenesis, including changes in p53, KRAS, CDH1, cyclin E, Her2, and MET [40]. The genetic alteration in p53 leading to the development of gastric cancer is also seen in *H. pylori*-associated conditions such as chronic gastritis, intestinal metaplasia, and dysplasia [41–43]. Intriguingly, p53 can also be negatively regulated by gankyrin in multiple cancer types [3, 10], which is similar to the observation in the current study that gankyrin involves the precancerous lesions of gastric cancer. To compare the oncogenic properties between cell lines, MKN45 (poorly differentiated adenocarcinoma) with high-gank expression seemed showing higher proliferation abilities than MKN74 (highly differentiated adenocarcinoma) with low-gank expression (Figures 2(a)–2(d)), which also hinted that gankyrin might promote the process of gastric cancer development. Thus, the data strongly suggested that gankyrin contributes to early malignant transformation and later processes of gastric cancer (Figures 1 and 2).

In a previous report, we demonstrated that gankyrin regulates the mTORC1 signaling pathway in CRC via a PI3K/AKT-independent and TSC-dependent mechanism [18]. On the other hand, the AKT activation was found to be involved in the gankyrin-induced mTORC1 signaling according to our findings in gastric cancer (Figure 3(a)), which indicated that gankyrin might be associated with or regulated by an alternative molecule to potentiate the mTORC1 signaling pathway. The analysis of the TCGA cancer genome database also revealed that the mRNA expression of gankyrin was clinically correlated to PGK1, an upstream protein kinase of AKT (Figure 3(b)). This evidence introduced a novel insight into gankyrin biology in gastric cancer. However, additional studies are essential to support that gankyrin activates PGK1/AKT signaling to enhance the mTORC1 activation. High ROS levels leading to oxidative stress limit cancer cell survival during certain windows of cancer initiation and progression [44, 45]. Recently, it has been reported that overexpressed gankyrin amplifies the antioxidant capacity of HCC cells, reduces oxidative stress-induced mitochondrial damage, inhibits apoptosis, and promotes the development of HCC [15]. Consistently, the present study demonstrated that gankyrin can significantly impede ROS through the activation of mTORC1 signaling in gastric cancer (Figure 4). Moreover, we found that gankyrin affected not only the production of hydrogen peroxide (Figures 4(d) and 4(e)) but also the superoxide anion (Figures 4(b) and 4(c)), which supplied an interesting cue that gankyrin may exert its function through superoxide anion. However, it still needs further study on it. Therefore, gankyrin might also exhibit effects of accelerating the cancer process by regulating oxidative stress and maintaining cell homeostasis through the mTORC1 signaling pathway.

## 5. Conclusions

In conclusion, the current study revealed that increased gankyrin expression could be a risk factor of harboring gastric cancer. It potentially drove malignant transformation and behaviors of gastric cancer cell, as well as alleviated oxidative stress through the mTORC1 pathway. These characteristics provide a new insight into gankyrin biology with respect to gastric cancer.

## Figures and Tables

**Table 1:** Gankyrin expression in noncancerous and precancerous gastric tissues.

Gankyrin	NAG	CAG	CAG + Im	CAG + Dys
Positive rate	2 (6.67%)	7 (23.33%)	24 (80.00%)	27 (90.00%)
Median (QR)	1 (1, 10)	30 (20, 45)^∗^	115 (80, 210)^∗∗^	150 (90, 236.25)^∗∗^

120 noncancerous and precancerous gastric tissues were analyzed for gankyrin expression. Positive gankyrin staining was defined as the H-score higher than the median 47.5. For each group, the median, 1st quartile, and 3rd quartile were shown. The *P* value was calculated by Mann–Whitney *U*-test vs. NAG (^∗^*P* < 0.05, ^∗∗^*P* < 0.01). NAG: nonatrophic gastritis; CAG: chronic atrophic gastritis; Im: intestinal metaplasia; Dys: dysplasia.

**Table 2:** Association between gankyrin expression and clinicopathological features.

Clinicopathological features	No. of patients (*n*)	Relative gankyrin expression		*T*	*P*
		High	Low		
Age				0.785	0.435
<45	9	5 (55.6%)	4 (44.4%)		
≥45	68	39 (57.4%)	29 (42.6%)		
Gender				1.898	0.062
Male	52	34 (65.4%)	18 (34.6%)		
Female	25	11 (44.0%)	14 (56.0%)		
Lymph node metastasis				2.401	0.019
No	20	9 (45.0%)	11 (55.0%)		
Yes	57	35 (61.4%)	22 (38.6%)		
Distant metastasis				2.532	0.013
No	55	27 (49.1%)	28 (50.9%)		
Yes	22	17 (77.3%)	5 (22.7%)		
Pathological grade				2.557	0.084
I–II	9	7 (77.8%)	2 (22.2%)		
III	62	34 (54.8%)	28 (45.2%)		
IV	6	3 (50.0%)	3 (50.0%)		
Vascular invasion				2.122	0.037
No	20	9 (45.0%)	11 (55.0%)		
Yes	57	35 (61.4%)	22 (38.6%)		

Patients were divided by high gankyrin staining (H-score ≥ 145) and low gankyrin staining (H-score < 145).

## Data Availability

The datasets analyzed during the current study are available in the TCGA (https://cancergenome.nih.gov/), GEO (https://www.ncbi.nlm.nih.gov/gds), PrognoScan dataset (http://www.prognoscan.org/), and HPA dataset (http://www.proteinatlas.org/). All data generated during this study are included in this article.

## References

[1] Padmanabhan B., Adachi N., Kataoka K., Horikoshi M. (2004). Crystal structure of the homolog of the oncoprotein gankyrin, an interactor of Rb and CDK4/6. *The Journal of Biological Chemistry*.

[2] Krzywda S., Brzozowski A. M., al-Safty R. (2003). Crystallization of gankyrin, an oncoprotein that interacts with CDK4 and the S6b (rpt3) ATPase of the 19S regulator of the 26S proteasome. *Acta Crystallographica. Section D, Biological Crystallography*.

[3] Krzywda S., Brzozowski A. M., Higashitsuji H. (2004). The crystal structure of gankyrin, an oncoprotein found in complexes with cyclin-dependent kinase 4, a 19 S proteasomal ATPase regulator, and the tumor suppressors Rb and p53. *The Journal of Biological Chemistry*.

[4] Higashitsuji H., Higashitsuji H., Itoh K. (2005). The oncoprotein gankyrin binds to MDM2/HDM2, enhancing ubiquitylation and degradation of p53. *Cancer Cell*.

[5] Liu Y., Zhang J., Qian W. (2014). Gankyrin is frequently overexpressed in cervical high grade disease and is associated with cervical carcinogenesis and metastasis. *PLoS One*.

[6] Kim Y. H., Kim J. H., Choi Y. W. (2013). Gankyrin is frequently overexpressed in breast cancer and is associated with ErbB2 expression. *Experimental and Molecular Pathology*.

[7] Valanejad L., Lewis K., Wright M. (2017). FXR-gankyrin axis is involved in development of pediatric liver cancer. *Carcinogenesis*.

[8] Riahi M. M., Sistani N. S., Zamani P., Abnous K., Jamialahmadi K. (2017). Correlation of gankyrin oncoprotein overexpression with histopathological grade in prostate cancer. *Neoplasma*.

[9] Umemura A., Itoh Y., Itoh K. (2008). Association of gankyrin protein expression with early clinical stages and insulin-like growth factor-binding protein 5 expression in human hepatocellular carcinoma. *Hepatology*.

[10] Higashitsuji H., Liu Y., Mayer R. J., Fujita J. (2005). The oncoprotein gankyrin negatively regulates both p53 and RB by enhancing proteasomal degradation. *Cell Cycle*.

[11] Li J., Tsai M. D. (2002). Novel insights into the INK4-CDK4/6-Rb pathway: counter action of gankyrin against INK4 proteins regulates the CDK4-mediated phosphorylation of Rb. *Biochemistry*.

[12] Dawson S., Higashitsuji H., Wilkinson A. J., Fujita J., Mayer R. J. (2006). Gankyrin: a new oncoprotein and regulator of pRb and p53. *Trends in Cell Biology*.

[13] Lozano G., Zambetti G. P. (2005). Gankyrin: an intriguing name for a novel regulator of p53 and RB. *Cancer Cell*.

[14] Bhattarai G., Lee Y. H., Lee N. H., Yun J. S., Hwang P. H., Yi H. K. (2011). C-myb mediates inflammatory reaction against oxidative stress in human breast cancer cell line, MCF-7. *Cell Biochemistry and Function*.

[15] Yang C., Tan Y. X., Yang G. Z. (2016). Gankyrin has an antioxidative role through the feedback regulation of Nrf2 in hepatocellular carcinoma. *The Journal of Experimental Medicine*.

[16] Chen Y., Li H. H., Fu J. (2007). Oncoprotein p28^GANK^ binds to RelA and retains NF-*κ*B in the cytoplasm through nuclear export. *Cell Research*.

[17] Ren Y. B., Luo T., Li J. (2015). p28^GANK^ associates with p300 to attenuate the acetylation of RelA. *Molecular Carcinogenesis*.

[18] Qin X., Wang X., Liu F. (2016). Gankyrin activates mTORC1 signaling by accelerating TSC2 degradation in colorectal cancer. *Cancer Letters*.

[19] Lozano R., Naghavi M., Foreman K. (2012). Global and regional mortality from 235 causes of death for 20 age groups in 1990 and 2010: a systematic analysis for the Global Burden of Disease Study 2010. *Lancet*.

[20] Chang A. H., Parsonnet J. (2010). Role of bacteria in oncogenesis. *Clinical Microbiology Reviews*.

[21] Gonzalez C. A., Sala N., Rokkas T. (2013). Gastric cancer: epidemiologic aspects. *Helicobacter*.

[22] Theodoratou E., Timofeeva M., Li X., Meng X., Ioannidis J. P. A. (2017). Nature, nurture, and cancer risks: genetic and nutritional contributions to cancer. *Annual Review of Nutrition*.

[23] Strong V. E., Gholami S., Shah M. A. (2017). Total gastrectomy for hereditary diffuse gastric cancer at a single center: postsurgical outcomes in 41 patients. *Annals of Surgery*.

[24] Langner C. (2017). Hereditary gastric and pancreatic cancer. *Pathology*.

[25] Venerito M., Nardone G., Selgrad M., Rokkas T., Malfertheiner P. (2014). Gastric cancer--epidemiologic and clinical aspects. *Helicobacter*.

[26] Zamani P., Matbou Riahi M., Momtazi-Borojeni A. A., Jamialahmadi K. (2017). Gankyrin: a novel promising therapeutic target for hepatocellular carcinoma. *Artificial Cells, Nanomedicine, and Biotechnology*.

[27] Wang C., Cheng L. (2017). Gankyrin as a potential therapeutic target for cancer. *Investigational New Drugs*.

[28] Wang W. P., Yan X. L., Li W. M. (2015). Clinicopathologic features and prognostic implications of gankyrin protein expression in non-small cell lung cancer. *Pathology, Research and Practice*.

[29] Hwang J. A., Yang H. M., Hong D. P. (2014). Gankyrin is a predictive and oncogenic factor in well-differentiated and dedifferentiated liposarcoma. *Oncotarget*.

[30] Malik T. H., Sayahan M. Y., al Ahmed H. A., Hong X. (2017). Gastric intestinal metaplasia: an intermediate precancerous lesion in the cascade of gastric carcinogenesis. *Journal of the College of Physicians and Surgeons*.

[31] Granelli P., Bonavina L., Zennaro F., Segalin A., Siardi C. (1994). Intestinal metaplasia: what is its role in gastric carcinogenesis?. *Minerva Gastroenterologica e Dietologica*.

[32] Yu T., Zhao Y., Hu Z. (2017). MetaLnc9 facilitates lung cancer metastasis via a PGK1-activated AKT/mTOR pathway. *Cancer Research*.

[33] Qian X., Li X., Lu Z. (2017). Protein kinase activity of the glycolytic enzyme PGK1 regulates autophagy to promote tumorigenesis. *Autophagy*.

[34] Halliwell B. (2007). Oxidative stress and cancer: have we moved forward?. *The Biochemical Journal*.

[35] Handa O., Naito Y., Yoshikawa T. (2013). Redox biology and gastric carcinogenesis: the role of *helicobacter pylori*. *Redox Report*.

[36] Correa P. (1992). Human gastric carcinogenesis: a multistep and multifactorial process--first American Cancer Society award lecture on cancer epidemiology and prevention. *Cancer Research*.

[37] Pittayanon R., Rerknimitr R., Klaikaew N. (2017). The risk of gastric cancer in patients with gastric intestinal metaplasia in 5-year follow-up. *Alimentary Pharmacology & Therapeutics*.

[38] Spence A. D., Cardwell C. R., McMenamin Ú. C. (2017). Adenocarcinoma risk in gastric atrophy and intestinal metaplasia: a systematic review. *BMC Gastroenterology*.

[39] Choi A. Y., Strate L. L., Fix M. C. (2018). Association of gastric intestinal metaplasia and East Asian ethnicity with the risk of gastric adenocarcinoma in a U.S. population. *Gastrointestinal Endoscopy*.

[40] Grabsch H. I., Tan P. (2013). Gastric cancer pathology and underlying molecular mechanisms. *Digestive Surgery*.

[41] Deng N., Goh L. K., Wang H. (2012). A comprehensive survey of genomic alterations in gastric cancer reveals systematic patterns of molecular exclusivity and co-occurrence among distinct therapeutic targets. *Gut*.

[42] Sue S., Shibata W., Maeda S. (2015). *Helicobacter pylori*-induced signaling pathways contribute to intestinal metaplasia and gastric carcinogenesis. *BioMed Research International*.

[43] Salih B. A., Gucin Z., Bayyurt N. (2013). A study on the effect of Helicobacter pylori infection on p53 expression in gastric cancer and gastritis tissues. *Journal of Infection in Developing Countries*.

[44] Gill J. G., Piskounova E., Morrison S. J. (2017). Cancer, oxidative stress, and metastasis. *Cold Spring Harbor Symposia on Quantitative Biology*.

[45] Vallejo M. J., Salazar L., Grijalva M. (2017). Oxidative stress modulation and ROS-mediated toxicity in cancer: a review on *in vitro* models for plant-derived compounds. *Oxidative Medicine and Cellular Longevity*.

